# Toward protein NMR at physiological concentrations by hyperpolarized water—Finding and mapping uncharted conformational spaces

**DOI:** 10.1126/sciadv.abq5179

**Published:** 2022-08-05

**Authors:** Ludovica M. Epasto, Kateryna Che, Fanny Kozak, Albina Selimovic, Pavel Kadeřávek, Dennis Kurzbach

**Affiliations:** ^1^ University of Vienna, Faculty of Chemistry, Institute of Biological Chemistry, Währinger Str. 38, 1090 Vienna, Austria.; ^2^ Masaryk University, CEITEC, Kamenice 5, 625 00 Brno, Czech Republic.

## Abstract

Nuclear magnetic resonance (NMR) spectroscopy is a key method for determining the structural dynamics of proteins in their native solution state. However, the low sensitivity of NMR typically necessitates nonphysiologically high sample concentrations, which often limit the relevance of the recorded data. We show how to use hyperpolarized water by dissolution dynamic nuclear polarization (DDNP) to acquire protein spectra at concentrations of 1 μM within seconds and with a high signal-to-noise ratio. The importance of approaching physiological concentrations is demonstrated for the vital MYC-associated factor X, which we show to switch conformations when diluted. While in vitro conditions lead to a population of the well-documented dimer, concentrations lowered by more than two orders of magnitude entail dimer dissociation and formation of a globularly folded monomer. We identified this structure by integrating DDNP with computational techniques to overcome the often-encountered constraint of DDNP of limited structural information provided by the typically detected one-dimensional spectra.

## Teaser

NMR signal boosters combined with molecular dynamics decipher protein structures at physiological concentrations.

## INTRODUCTION

Nuclear magnetic resonance (NMR) spectroscopy is a key method for determining the structures and dynamics of proteins and nucleic acids (*1*–*3*). Currently, NMR is the only method that enables complete structural characterization of biomacromolecules with atomistic resolution in their native solution environment. However, because of the intrinsically low sensitivity of the method, the target molecule’s concentration needs to be much higher than under typical physiological conditions. This is crucial because interaction kinetics, complex structures, and accessible conformational spaces are often concentration dependent (*4*–*7*). As a result, typical in vitro conditions limit the relevance of NMR data. In contrast, developing NMR methods that enable access to the low micromolar physiological concentration regime can help to improve the medicinal relevance of NMR and thus widen the scope of its applications. Such developments remain challenging, even despite extensive and continuous efforts, such as developing costly ultrahigh-field magnets and cryogenically cooled probes (*8*).

Here, we demonstrate an unconventional route toward overcoming the NMR concentration limitation. We show how to record NMR spectra of proteins within seconds at micromolar concentration and a signal-to-noise ratio (SNR) > 15 by dissolving them in “hyperpolarized water” (*9*–*17*). This means that the NMR signals of the water protons are enhanced in comparison to conventional NMR by pretreating it in a dedicated DNP (dynamic nuclear polarization) apparatus before being used for dissolving the target protein. Other applications of hyperpolarized water can, for example, be found in magnetic resonance imaging (*18*, *19*) or low-field (triplet or Overhauser DNP) NMR (*20*–*26*). The hyperpolarization boosts the water NMR signal intensities by more than two orders of magnitude. Through exchange interactions between the water and the target biomolecule, the signals of the latter are similarly boosted by orders of magnitude, hence enabling access to equivalently >100-fold reduced concentration regimes (*9*–*17*).

The value of this methodological advance is demonstrated using the ubiquitous transcription factor MAX (MYC-associated factor X; *M*_w_ = 10.9 kDa per monomer) (*27*–*32*). We show that MAX adopts an undocumented conformation when concentrations approach physiological levels (*33*) in contrast to the high-concentration regime typically encountered in in vitro assays (*32*–*34*). By combining the hyperpolarization methodology with molecular dynamic (MD) simulations, chemical shift prediction, and established structural biology methods, we characterized the newly found MAX conformation in atomistic detail.

This is relevant because the vital dimerization of MAX with its partner molecule MYC inevitably depends on MAX’s conformation (*5*, *35*). The importance of this process can be assessed considering that the MYC:MAX heterodimer plays a crucial role in cell proliferation and apoptosis (*31*) and that its dysregulation is associated with a plethora of cancers (*31*).

MAX is typically described as a stably folded homodimer in solution. To bind to MYC, the homodimer yet needs to dissociate. Despite its significance, the question of how the process of MAX homodimer destabilization proceeds remains unanswered, not least because of a strong dimerization tendency and nanomolar homomolecular affinities (*33*). To tackle this dilemma, we map a formerly uncharted part of MAX’s conformational space, i.e., the regime at physiological concentrations, and characterize the structures found therein with DNP-boosted NMR. This might help shed light on the MYC:MAX dimerization enigma from the viewpoint of the MAX monomer.

## RESULTS

**Fig. 1.**
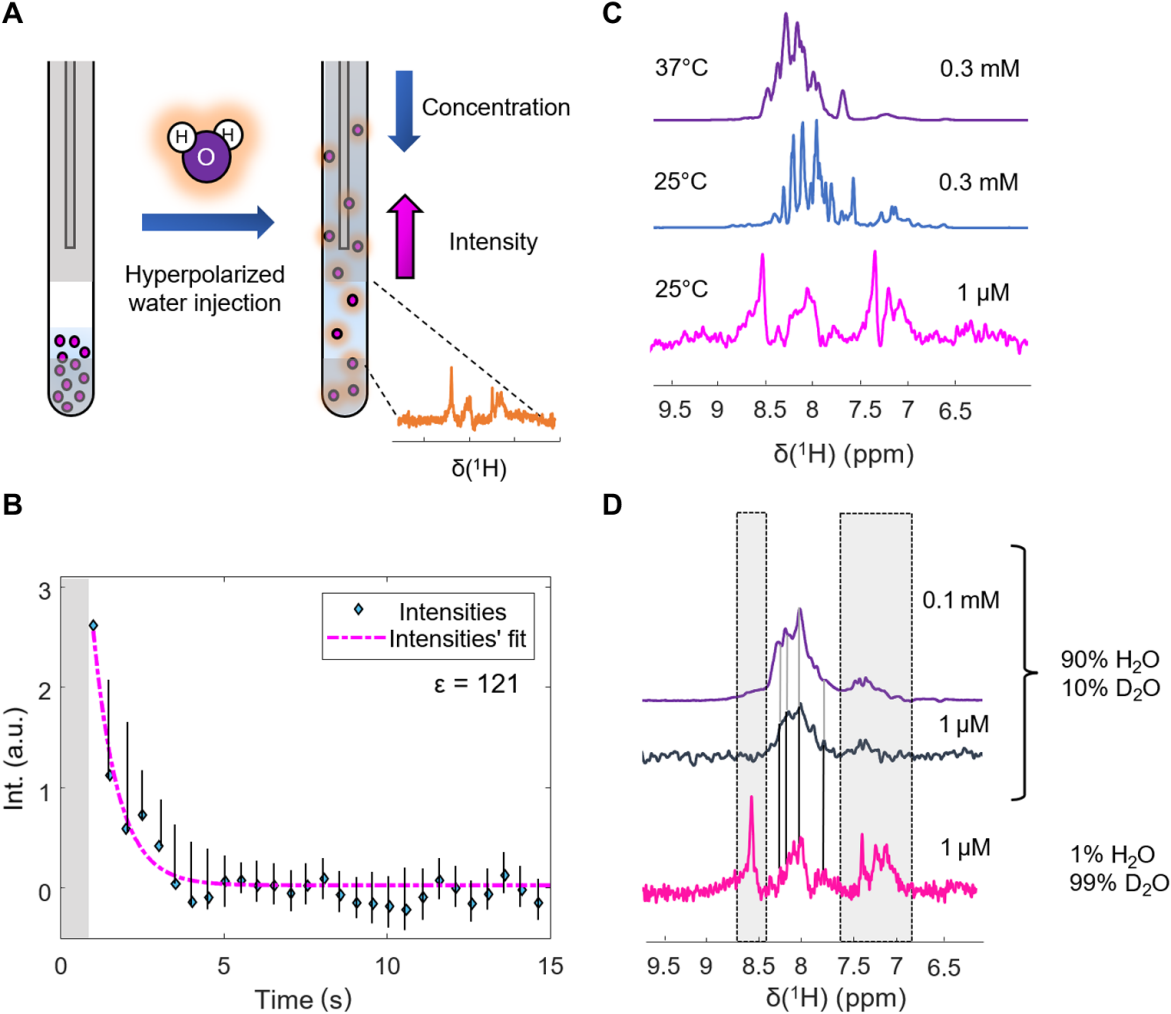
NMR and DDNP of MAX. (**A**) Sketch of the experimental concept. Upon injection of hyperpolarized water, the target protein is diluted, while at the same time, the signal loss is (over)compensated by exchange between the water and the target, which introduces hyperpolarized protons into the latter. (**B**) Signal decay (bulk integrals) of the ^1^H^N^ resonance of MAX after dilution. Directly after injection, turbulences obscure the spectra (gray bar). After 1 s, the turbulences settle, and signal-amplified spectra become detectable. After 10 s, the signal amplitude has decayed below the noise level. The experiment was repeated two times. The error bars indicate the maximum deviation between the repetitions. (**C**) Comparison of skyline projections on the ^1^H dimension of an HSQC (in purple) and a TROSY (in blue) experiment detecting ^15^N-labeled MAX (0.3 mM) with a hyperpolarized MAX spectrum (first detected spectrum) at near-physiological concentration (1 μM). The latter was recorded using an adapted BEST-HMQC pulse sequence. The spectra at higher concentrations can be considered fingerprints of the folded dimer (25°C) and the unfolded state (37°C). The spectrum at low concentrations matches neither of these cases, indicating the presence of a third conformational state. (**D**) ^15^N-edited ^1^H spectra of ^15^N-labeled MAX at different dilutions detected on an 11.7-T NMR spectrometer in thermal equilibrium (purple and blue) compared to the hyperpolarized ^15^N-edited ^1^H spectrum (magenta; average over the first 20 detected spectra). The black straight lines trace the position of significative peaks from the thermal equilibrium to the hyperpolarized spectrum. The dashed boxes indicate significantly boosted signals in the NMR experiment that remained undetected in the thermal equilibrium spectrum. The DDNP experiment highlights almost invisible signals in the spectral envelope obtained in the standard experiments. a.u., arbitrary units.

To obtain NMR spectra of MAX at physiological concentrations in hyperpolarized water, we used dissolution DNP (DDNP) (*36*–*38*). We followed the protocol detailed in (*39*, *40*). In brief, hyperpolarized water was produced in a dedicated DNP apparatus under cryogenic conditions (*T*_DNP_ = 1.3 K) by microwave irradiation of dissolved stable radicals [15 mM TEMPOL (4-Hydroxy-2,2,6,6-tetramethylpiperidinyloxyl]. To this end, we used the home-built prototype described by Kress *et al.* (*41*). After polarization buildup, the hyperpolarized water was rapidly heated to ambient temperatures and pneumatically transferred to an NMR spectrometer where a MAX solution was waiting “in situ” in the NMR tube. The hyperpolarized water was then mixed with the MAX solution (0.3 mM), diluting it 300-fold before the NMR acquisition (Fig. 1A). Upon detection, the final concentration was 1 μM. The entire dissolution and mixing process took ca. 2 s.

Upon mixing, the proton spin hyperpolarization of the water is spontaneously transferred to the target protein through chemical exchange and nuclear Overhauser effects, boosting the signal amplitudes of the target (*17*). A series of ^15^N-edited ^1^H one-dimensional (1D) NMR spectra was then recorded for 120 s with a sampling rate of 2 s^−1^ [the pulse sequence was similar to the first increment of a BEST-HMQC (band-selective short transient heteronuclear multiquantum correlation) (*42*); see fig. S1].

The resulting signal intensity as a function of time is shown in Fig. 1B. An intense spectrum can be observed directly after mixing. It decays to thermal equilibrium within ~10 s. The initial SNR was found to be >15. The typical comparison to thermal equilibrium data was complicated because not all peaks were visible in the nonhyperpolarized spectra after decay to thermal equilibrium. The enhancement of the bulk signal was ca. 120 (see fig. S2 for the spectrum in thermal equilibrium). It should be noted that the data were recorded on a 500-MHz spectrometer equipped with a broadband probe, which is designed for the detection of heteronuclei. Much better SNR could be obtained with a probe dedicated to ^1^H detection and higher magnetic fields. Other targets, particularly intrinsically disordered proteins, that typically feature sharper NMR signals (*11*) might even further push the detection limit.

Concerning the experimental times, one must consider that the buildup of water hyperpolarization takes ca. 2 hours (*16*), while the detection only takes a few minutes. Taking the preparation of the dissolution experiments into account, too, the time needed is comparable to that of a conventional NMR experiment recorded via signal averaging. However, as the gain in signal amplitude is >120 for the case at hand, >14,000 signal averages would be required to reach a similar SNR.

Figure 1C shows a comparison of the spectrum obtained in hyperpolarized water with spectra recorded by conventional NMR. Notably, the hyperpolarized spectrum obtained at physiological concentrations differs from the other spectra recorded under typical in vitro NMR conditions. In particular, we compared the DDNP-derived spectrum obtained at a concentration of 1 μM with HSQC (heteronuclear single-quantum coherence) and TROSY (transverse relaxation–optimized spectroscopy) spectra, obtained at a concentration of 0.3 mM at 37° and 25°C, respectively. Under these conditions, MAX populates one of two well-documented conformations, a stably folded helical homodimer (*43*) at 25°C and a monomeric random-coil state (*29*) at 37°C (the elongated dimer tumbles slowly around one axis, necessitating TROSY). Evidently, the low-concentration spectrum matches neither of these two cases. Hence, a third conformation in an undocumented conformational subspace seems to be populated when concentrations approach physiological levels, independent of the environmental temperature.

In the next step, we confirmed this conclusion by comparing the hyperpolarized spectra with references in thermal equilibrium recorded with the same real-time ^15^N-edited ^1^H 1D BEST-HMQC detection sequence. The result is shown in Fig. 1D. Two experiments were carried out at 0.1 mM and 1 μM. It is evident that the conformation populated at low concentration leads to a spectrum significantly different from the one recorded at higher concentration. Only upon reducing the concentration to 1 μM, the features of the reference spectrum can be observed in the hyperpolarized spectrum, too (see fig. S3 for a more detailed representation). It should be noted that with hyperpolarization, some signals can be observed that remained below the detection threshold in the thermal equilibrium case, particularly in the regions around 8.5 and 7.5 parts per million (ppm) (Fig. 1D, gray boxes).

These resonances are significantly stronger enhanced (compared to thermal equilibrium) than other parts of the spectrum. An analysis of the predicted chemical shifts (cf. table S1) showed that these resonances belong exclusively to Q-, D-, N-, and H-type residues. These amino acids all feature labile side-chain protons, and it was recently shown (*16*, *40*) that amino acids with such moieties display stronger signal enhancements in hyperpolarized water than amino acids void of exchangeable side-chain protons. It was concluded (*16*, *40*) that exchange-relayed nuclear Overhauser magnetization transfers from the side chains to the detected amides additionally boost these resonances. A similar effect likely underlies the data presented in Fig. 1D, too. Similar reasoning may account for the substantial enhancements of the side-chain resonances between 7 and 7.5 ppm of N and Q residues.

Hence, we conclude from the data in Fig. 1 that the DDNP results show a spectral fingerprint of MAX in a region of its conformational space that is dominated neither by the MAX homodimer (high concentrations, low temperature) nor by its random-coil state (high concentrations, high temperatures). HSQC experiments in thermal equilibrium further support this (see figs. S4 and S5), showing, despite low SNR, that spectra obtained at near-physiological concentrations match neither that of the folded nor that of the intrinsically disordered state.

We want to stress that data can be recorded at concentrations of 1 μM in thermal equilibrium (in particular when using cryogenically cooled probes), but important features/peaks of the spectra might be overlooked as these remain below the sensitivity threshold. Contrary, with DDNP, weak signals can be recovered that vividly exchange with the solvent, such as those of the Q-, D-, N-, and H-type residues.

Note that damage of MAX due to the injection of hyperpolarized water is unlikely. Control DDNP experiments at higher concentration (fig. S6) and thermal equilibrium spectra of reconcentrated MAX after a DDNP experiment in a radical-free buffer all led to NMR spectra of the intact MAX dimer (see fig. S7).

**Fig. 2.**
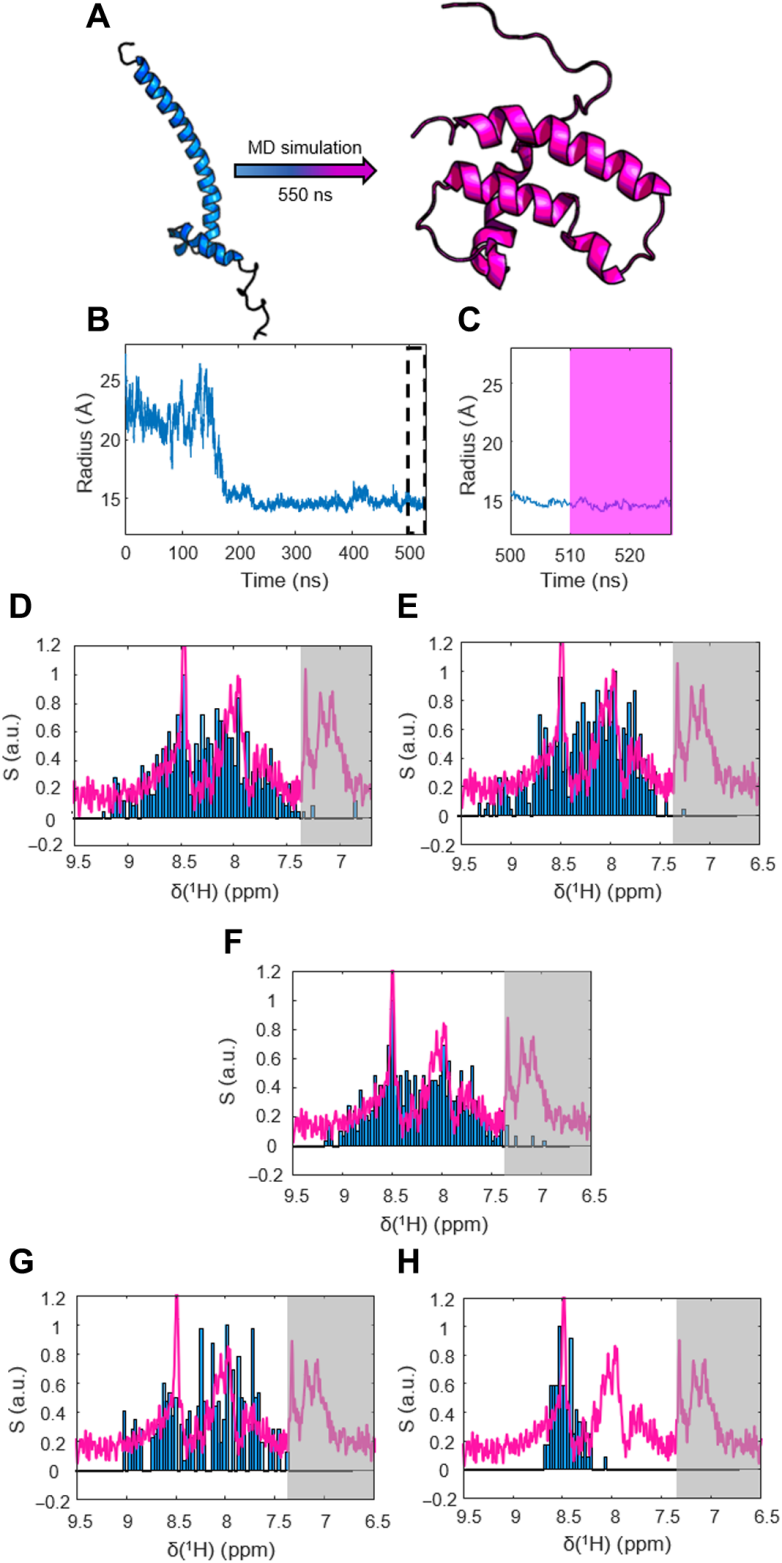
Integration of MD simulations with the DDNP spectra. (**A**) Structural transition observed for the MAX monomer in a 550-ns MD simulation in a 15 nm^3^ large box. Stripping the well-documented dimer of one subunit provided the starting structure. After 150 ns, a transition into a globular, tightly folded conformation could be observed. (**B**) Development of the hydration radius *R*_h_ of MAX in the MD simulations over time. After ~150 ns, MAX converts from the initial elongated shape into a compact conformation. The dashed box indicates the part of the simulation that we considered equilibrated and used for chemical shift predictions. (**C**) Zoom onto the *R*_h_ trajectory and the last 30 ns of the simulation. All conformations used for chemical shift prediction stem from the last 20 ns of simulation, highlighted in pink. (**D** to **F**) Comparison of the hyperpolarized spectrum (S) (pink line; average over the first 20 detected spectra) and the spectrum predicted on the basis of the MD simulations (blue bars) of the MAX monomer for three independent runs. The gray shade indicates side-chain resonances not included in the chemical shift calculations. The good match between the experimental and the calculated spectrum supports our conclusion that the monomer dominates the conformational ensemble at low concentrations. (**G**) “Negative control” comparing the experimental hyperpolarized spectrum with a spectrum predicted from the elongated MAX dimer structure. The mismatch shows that the experiment does not reflect this structure. (**H**) Negative control comparing the experimental hyperpolarized spectrum with a spectrum predicted for a random-coil state. The mismatch shows that the experiment does not reflect this structure either.

An intuitive explanation for the conformational switch is a concentration-dependent monomer-to-dimer ratio and that the monomer dominates the conformational space at low concentrations. However, neither conventional NMR nor crystallography can be used to resolve the structural dynamics of monomeric MAX at such high dilution. We, therefore, used MD simulations to identify a possible monomeric MAX species. Because of the lack of a known monomer structure, we selected one chain from the NMR solution structure of the homodimer reported in the literature (*32*) as starting point [this choice was supported by structure prediction through the PHYRE2 software (*44*), which yielded a similar conformation for the MAX monomer; see fig. S8]. We then ran MD simulations of the monomer in explicit solvent for >500 ns. The result is shown in Fig. 2 (A to C) (for details, see the Materials and Methods). The initially elongated monomer rapidly switches its conformation to a globular shape with stable secondary and tertiary structural elements (Fig. 2A). The major structural elements of MAX, i.e., the helix-loop-helix motif, are retained, yet the N-terminal helix that typically forms the leucine zipper (LZ) domain folds back toward the N terminus. Considering that the N and C termini feature opposite charges (MAX is one of the strongest dipoles found in nature) (*45*), such a configuration is likely energetically favorable in the absence of a second unit to form a dimer and constitute the LZ. In our simulations, this transition was evidenced by a sudden reduction in hydrodynamic radius (Fig. 2B), which drops from 2.5 to 1.5 nm upon transition from the elongated to the globular state. Once globularly folded, MAX did not return to the initial state in our simulation. The result was reproduced in three independent runs (see figs. S9 to S13).

To integrate the DDNP and MD data, we then used the Sparta+ software package (*46*) to calculate the ^1^H chemical shifts of the newly found globularly folded structure. We randomly chose 16 conformations from the last 20 ns (Fig. 2C) of the MD trajectory and predicted the amide ^1^H^N^ chemical shift (see table S1). Computing histograms of the chemical shift distribution (combining all 16 datasets) then yielded predictions of the ^15^N-edited ^1^H spectra. The results for the three independent simulations are shown in Fig. 2 (D to F). The measured hyperpolarized spectra and predicted ^15^N-edited ^1^H spectra match well within the precision of the approach. The resemblance of experimental and predicted data strongly supports our conclusion that a formerly undocumented conformation of a MAX monomer exists in lowly concentrated solutions. The match suggests that the globularly folded MAX monomer found in the MD simulations represents the conformation detected by DDNP-boosted NMR at physiological concentrations.

Note that the simulations do not consider differential line broadening of individual residues or the dependence of the individual residue signal intensities on the site-specific effectiveness of solvent exchange in the DDNP experiments. This could lead to mismatches between experimental and simulated results and data misinterpretation even for similar protein conformations. Therefore, it is important to perform negative control experiments and compare the DDNP-derived spectra to known cases. For MAX, the unfolded state, as well as the coiled-coil heterodimer, can be chosen as control structures.

We predicted the ^15^N-edited ^1^H spectra for the MAX dimer, using the available NMR solution structure as input for Sparta+ and a random-coil state of MAX, using neighborhood-corrected chemical shift prediction (Fig. 2, G and H) (*47*). In both cases, the experimental spectrum was not reproduced, supporting our conclusion that the hyperpolarized water–boosted NMR experiments detected a globularly folded MAX monomer.

Both control cases do not suffice to explain our results, although these two conformations are the primary constituents of the (so far documented) MAX conformational space [see, e.g., the work of Fieber *et al.* (*33*)]. Hence, necessarily, the DDNP experiments show a third, so far unknown, conformation.

The number and type of required control experiments depend on the detail of information that one wants to extract from the DDNP-enhanced spectra. Here, we compare our simulations to the overall experimental line shape, i.e., we refrain from residue-resolved data analysis. For this type of analysis, the two reported controls are sufficient as both clearly do not match the experimental line shape and width.

## DISCUSSION

In conclusion, we find that hyperpolarized water can boost the sensitivity of protein NMR spectra to access the low–micromolar concentration regime at high SNR and within short experimental times (acquisition in less than 1 min). This is possible even for a challenging target such as MAX, which is typically studied by NMR at concentrations of >1 mM (*32*). This enabled the identification and characterization of globularly folded monomeric MAX.

The predominance of a globular conformation at low concentrations is independently supported by fluorescence resonance energy transfer data reported by Vancraenenbroeck and Hofmann (*48*). They showed that at very low concentrations, monomeric globular conformations dominate the conformational space of MAX. In contrast, the coiled-coil conformation of the MAX dimer features a strongly anisotropic shape, which could only be found at higher concentrations. However, this study focused on kinetic analyses and did not report a resolved conformational model at low concentrations. Our combined DDNP/MD approach yet can provide atomistic protein structures to map the conformational spaces at high detail. In the broader context of cellular transcription, the monomer concentration of unbound MAX in the cell nucleus is of particular interest as it might be directly involved in the dimerization rate with the MYC transcription factor.

It should be noted, however, that the presence of DNA in the cell nucleus, where MAX resides in vivo, favors the population of the homodimer because of the high stability of the MAX:MAX-DNA complex (*33*). Under in vivo conditions, MAX remains mainly in this form. The concentration of unbound MAX might therefore be much lower than 1 μM.

## MATERIALS AND METHODS

### Protein production

His-tagged MAX was subcloned into a pET-21a(+) expression vector and transformed into *Escherichia coli* Rosetta2 cells. The bacteria were grown at 37°C in M9 [for ^15^N labeling, ^15^N ammonium chloride (1 g/liter) was added] and induced at an optical density corresponding to *A*_600_ (absorbance at 600 nm) = 0.6 with 1 mM isopropyl-β-d-thiogalactopyranoside before incubation at 30°C overnight. Cell pellets were homogenized in 25 mM tris, 100 mM NaCl, and 2 mM β-mercaptoethanol (pH 8.00). After cell disruption, the supernatant was purified through Ni^+^ affinity chromatography. The his-tag was cleaved by Tobacco Etch Virus (TEV) protease in a ratio of 1:15 to MAX at 4°C overnight. The cut was confirmed through mass spectrometry, reporting a mass of 11.035 kDa, consistent with the expected ^15^N-enriched protein mass (see fig. S14).

The sample was buffer-exchanged using ultracentrifugal filters with a cutoff of 3 kDa into the buffer used for the NMR experiments [25 mM MES, 25 mM NaCl, and 100 mM ArgHCl (pH 5.5)]. Last, MAX was concentrated up to 0.3 mM and aliquoted.

### Dissolution dynamic nuclear polarization

For DNP, 150 μl of a solution of 15 mM TEMPOL in a mixture of 50% glycerol-d_8_, 40% D_2_O, and 10% H_2_O was hyperpolarized at 1.4 K in a magnetic field of 6.7 T for 2500 s using continuous-wave microwave irradiation at 188.08 GHz. DNP samples were always freshly prepared to avoid ripening effects (*49*). A Virginia Diodes Inc. (VDI) microwave source was used together with a 16× frequency multiplier that provided an output power for the microwave of ca. 50 mW. The magnet-cryostat combination was purchased from Cryogenic Ltd. and operated as described in (*41*).

For detection of the solid-state polarization, a 400-MHz Bruker Avance III system was adapted to a ^1^H resonance frequency of 285.3 MHz and a ^13^C frequency of 71.72 MHz by using a broadband preamplifier for both channels. The detection circuit and the external tune-and-match system were home-built, as described in (*50*). To monitor the buildup, detection pulses with a flip angle of 1° were applied every 5 s.

After DNP, the sample was dissolved with a burst of 5 ml of D_2_O at 1.5 MPa as described in (*41*). The hyperpolarized liquid was then pushed with helium gas at 0.7 MPa to the detection spectrometer. The dissolution process used a home-built pressure heater actuated with an Arduino microcontroller. A home-written MATLAB-based user interface controls the dissolution and injection steps.

Detection was carried out using a 500 MHz Bruker NEO spectrometer equipped with a Prodigy BBFO cryogenic probe. The pulse sequence for detection corresponded to a series of detections based on the first increment of a BEST-HMQC (see fig. S1) (*42*). We used PC9 and RSNOB (*51*) selective 90° and 180° pulses covering a bandwidth of 4 ppm centered around a carrier frequency of 9.5 ppm to excite and invert the protons. The pulse lengths were 3495 and 1165 μs, respectively. The 90° pulse for the ^15^N channel was 15 μs long. The ^15^N carrier frequency was adjusted to 118 ppm. Heteronuclear decoupling was achieved using the GARP (*52*) scheme as preinstalled in Bruker TOPSPIN 4. The delay *d*_0_ was fixed to 0.1 μs. Note that varying *d*_0_ between 0 and 0.2 μs does not significantly affect the recorded spectra.

For sample injection, a “syringe” device similar to the one by Kouril *et al.* (*53*), but adapted to liquids, was used that injects a controlled volume into the NMR tube (see fig. S15 for a sketch of the setup). It should be noted that protein samples are often showing temperature-dependent conformational changes. For the dissolution system used here, the hyperpolarized water arrived with a temperature of 24° to 26°C in the NMR tube waiting in the spectrometer at 25°C (as controlled by the built-in temperature control system of the Bruker NEO spectrometer), such that no strong temperature gradients biased the protein conformation. However, other DNP systems that use other dissolution systems might lead to different sample temperatures.

To perform DDNP experiments with MAX at higher concentrations, the same procedure was followed, but MAX was only diluted by a factor of 3 during the injection, resulting in a final concentration of 0.1 mM upon detection (see fig. S6 for the results). For reproduction experiments, see fig. S16.

### Nuclear magnetic resonance

TROSY and HSQC spectra were recorded using a Bruker NEO 600-, 700-, or 850-MHz spectrometer equipped with cryogenically cooled probe heads optimized for ^1^H detection. Spectra were recorded in the States-TPPI/PFG mode for quadrature detection with carrier frequencies for ^1^H^N^ and ^15^N of 4.73 and 120.0 ppm, respectively. The samples contained 1 μM, 0.1 mM, or 0.3 mM MAX; 25 mM MES; 100 mM ArgHCl; and 25 mM NaCl (pH 5.5) in a 90% H_2_O/10% D_2_O mixture.

All NMR spectra were processed and analyzed using TOPSPIN 4.0.7 and MATLAB R2019a. NMRPipe and SPARKY were used to process and analyze the recorded TROSY and HSQC data (*54*, *55*). A squared and 60° phase-shifted sine bell window function was applied in all dimensions for apodization. Time-domain data were zero-filled to twice the dataset size, before Fourier transformation.

In addition, we considered the possibility that a shift in pH could influence MAX’s configuration. Therefore, we performed a TROSY experiment on MAX in a 30 times diluted buffer (fig. S5). Despite the significative variation of pH (from 5.5 to 7.15) in the lowly concentrated buffer, the detected spectrum does not show any similarities with the spectra obtained from a lowly concentrated protein sample (fig. S4C).

### MD simulations and chemical shift prediction

MD simulations were performed using the YASARA software package (*56*, *57*). The AMBER14 force field was used with periodic boundary conditions (*58*). Explicit water and a NaCl concentration of 0.9% were used for filling and neutralizing the simulation cell. Nonbonded interactions were cut off at 1.05 nm. Long-range Coulombic interactions were treated by a smoothed particle-mesh Ewald method (*59*). MD trajectories of >500-ns length were then accumulated. In total, three trajectories were computed. Intermolecular forces were recalculated at every second simulation substep. Temperature rescaling was used with a set temperature of 25°C. The box dimensions (cubic of >150-Å side length) were controlled to yield a solvent pressure of 1 bar. Snapshots of the simulations were taken every 4000 fs.

The chemical shifts were predicted from the MD simulations or the MAX dimer NMR structure (Protein Data Bank code 1R05) using the Sparta+ webserver (*46*). To this end, a set of 16 random snapshots from the last 20 ns of each MD trajectory was submitted for chemical shift prediction. The spectra were then calculated by computing averages over histograms (with 100 bins each) that represent the distribution of resonances over the δ(^1^H) dimension for each single snapshot. These tasks were performed using the MATLAB software package.

Neighborhood-corrected chemical shift calculations were carried out using the ncIDP web server (https://st-protein02.chem.au.dk/ncIDP/). These calculations predict chemical shifts (*47*) of unfolded proteins and peptides on the basis of their primary amino acid sequence. For calculating the chemical shift, a random-coil conformation is assumed for the protein or peptide. Database values for documented chemical shifts of all 20 amino acids in random-coil type structures are combined with sequence-dependent left- and right-neighbor (in terms of the primary sequence) correction factors. This results in an accurate prediction of the chemical shift for the protein when existing unfolded. Hence, this type of prediction can be used for the negative control in Fig. 2H (benchmark against unfolded MAX).

## Notes

View/request a protocol for this paper from *Bio-protocol*.
