# Supplementary material for: Toward protein NMR at physiological concentrations by hyperpolarized water—Finding and mapping uncharted conformational spaces
Source: Sci Adv. 2022 Aug 5;8(31):eabq5179. doi: 10.1126/sciadv.abq5179 (PMC9355353; doi:10.1126/sciadv.abq5179)
Supplement: Supplementary file 1 — Figs. S1 to S16 Table S1 [file sciadv.abq5179_sm.pdf]

Science Advances
AAAS

Supplementary Materials for

Toward protein NMR at physiological concentrations by hyperpolarized water—Finding and mapping uncharted conformational spaces

Ludovica M. Epasto *et al.*

Corresponding author: Dennis Kurzbach, dennis.kurzbach@univie.ac.at

Sci. Adv. **8**, eabq5179 (2022)
DOI: 10.1126/sciadv.abq5179

This PDF file includes:

Figs. S1 to S16
Table S1

Supporting Information

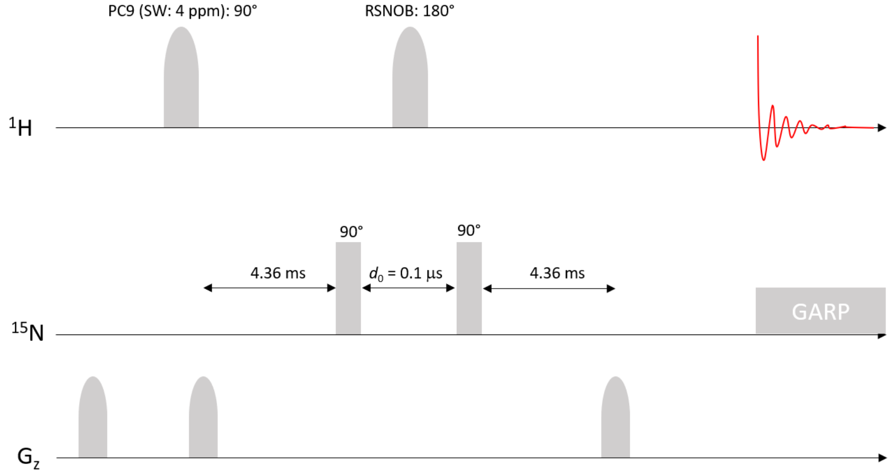

Figure S1. Pulse Sequence for DDNP Detection. Pulse sequence used for detection in the DDNP experiments. We used PC9 and RSNOB41 selective 90° and 180° pulses covering a bandwidth of 4 ppm centered around a carrier frequency of 9.5 ppm to excite and invert the protons. The pulse lengths were 3495 and 1165 μs, respectively. The 90° pulse for the ^{15}N channel was 15 μs long. Heteronuclear decoupling was achieved using the GARP42 scheme as preinstalled in Bruker TOPSPIN 4.

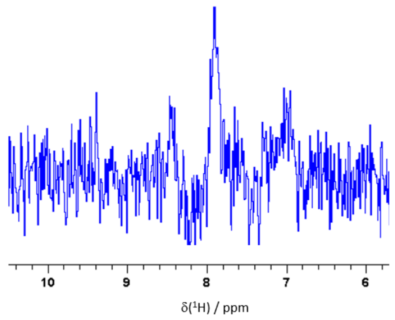

Figure S2. Thermal equilibrium spectrum. The thermal equilibrium spectrum recorded directly after the DDNP experiments in 98% deuterated buffer. This spectrum was used to calculate the signal enhancement.

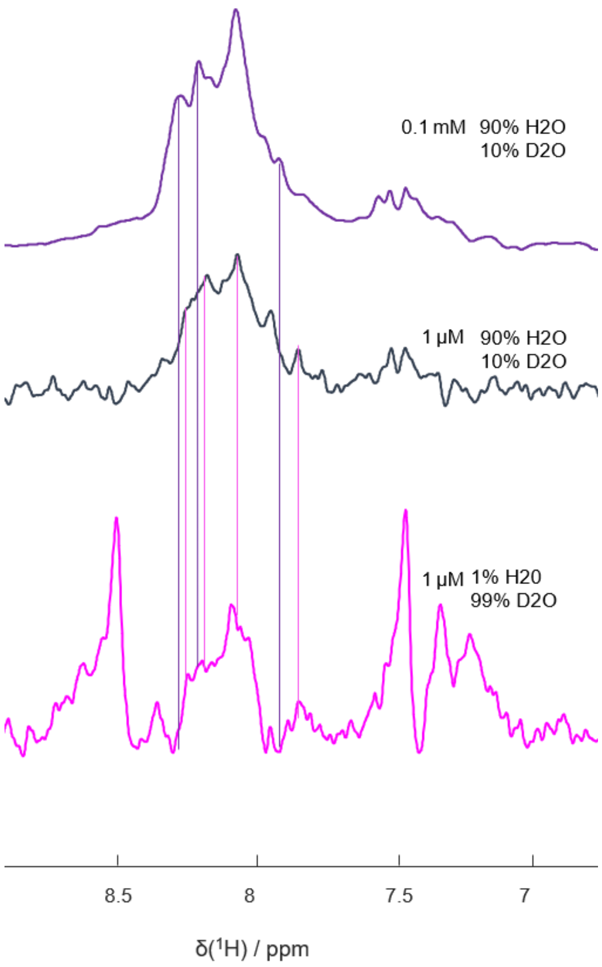

Figure S3. **Enlarged spectra.** Zoom onto the spectra of Fig. 1d. The magenta lines show similarities between the low concentration spectra, while the purple lines show differences between the high and low concentration spectra.

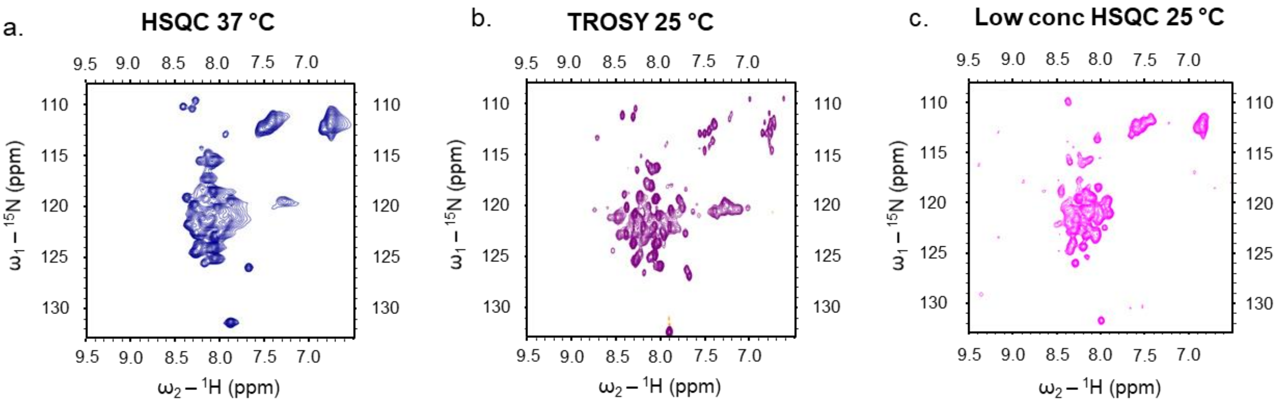

Figure S4. Thermal Equilibrium 2D NMR. MAX ^{15}N spectra at high concentration (0.3 mM) and low concentration (1 μM). a) HSQC experiment of 0.3 mM MAX ^{15}N collected at 37 °C on a 700 MHz Bruker NEO spectrometer. Most signals overlap, indicating that MAX mainly populates its unfolded state in these conditions. b) 0.3 mM MAX ^{15}N TROSY experiment at 25 °C. on a 700 MHz Bruker NEO spectrometer At this temperature, the folded conformation of MAX dominates the spectrum. c) HSQC spectrum of MAX ^{15}N at 1 μM recorded at 25 °C. The spectrum indicates a conformation that is different from both the spectra collected at high concentrations. Note that this spectrum was recorded on a high-field spectrometer (600 MHz) equipped with a lHe-cooled TXI cryogenically cooled probe (compared to a 500 MHz spectrometer equipped with a BBFO prodigy probe used for the DDNP experiments) to obtain a sufficiently high signal-to-noise ratio. In addition, we did not use the DNP-buffer (98% D_2O and contaminated with TEMPOL) but switched to a radical-free MES buffer in 90% H_2O/10% D_2O at pH 5.5. These changes significantly boosted the signal amplitudes in thermal equilibrium and enabled recording the spectrum. (Note that the samples for the thermal equilibrium spectra were prepared conventionally, such that pressure or temperature shocks can be excluded.)

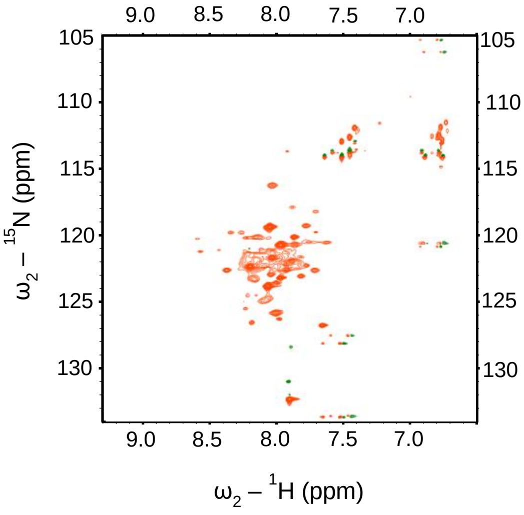

Figure S5. Influence of Buffer Strength. TROSY experiment on a 700 MHz Bruker NEO spectrometer of MAX ^{15}N at high concentration (0.3 mM) in 30-fold diluted MES buffer (0.83 mM NaCl, 0.83 mM MES, 3.33 mM ArgHCl in ddH_2O) performed on a 700 MHz Bruker spectrometer at 25 °C. Buffer effects can be excluded.

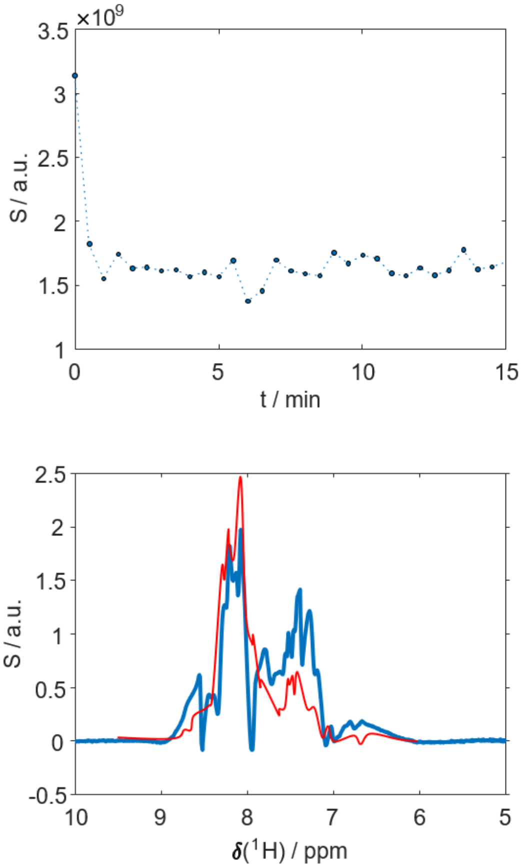

Figure S6. Reference DDNP Experiment at High Concentrations. Signal decay (top) and line shape (bottom) of MAX in hyperpolarized water at a final concentration of 0.1 mM. The elongated coiled-coil folded dimer does not recieve as high signal boosts from the solvent as the monomer likely due to the its short relaxation times. The spectrum (blue line, bottom) resembles that of the MAX dimer (red line) showing that injection of hyperpolarized water does not perturb the protein structure although the signal enhancement is not homoegenous across the spectrum (as expected when dissolving a protein in hyperpolarized water).

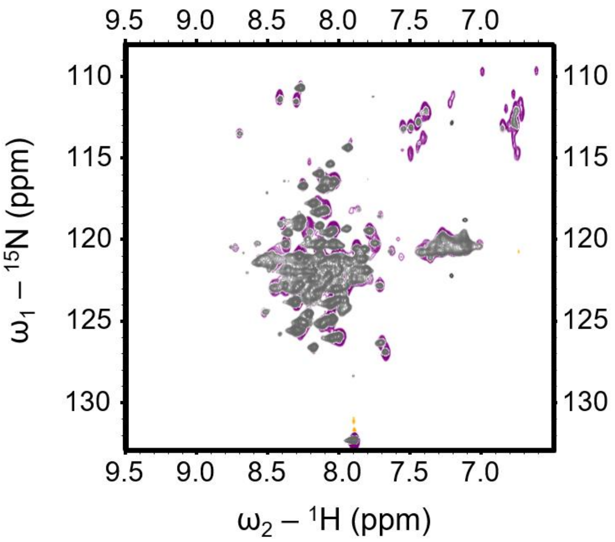

Figure S7. Protein Stability Check. Overlay of a ^{1}H-^{15}N TROSY of MAX at 0.1 mM in MES buffer at 25°C before (purple) and after reconcentration in TEMPOL-free MES after the dissolution experiment (grey). The spectra are identical showing that the protein wasn't damaged during the DDNP experiment.

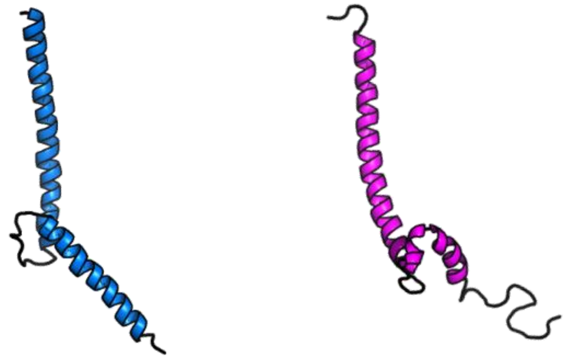

Figure S8. MD Starting Structure Validation. Comparison between the monomeric MAX structure predicted by Phyre2 (in blue) and the monomer extrapolated from the known structure of MAX dimer (in magenta). Despite slight differences at the N-terminus, both structures show the documented basic-helix-loop-helix-ZIP conformation.

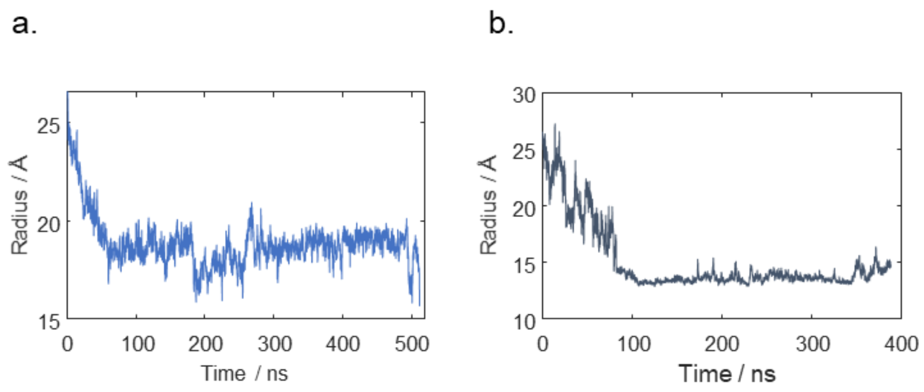

Figure S9. ***R_h* Trajectories.** Radius of hydration over time for the two MD replica (next to the data in Fig 2 of the main text).

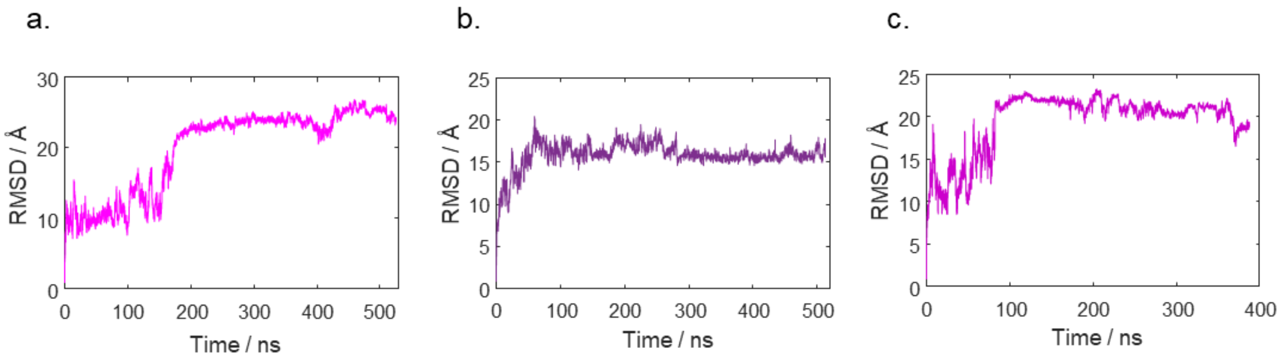

Figure S10. RMSD Trajectories. Average root-mean-square deviation from the starting structure coordinates of the first (a.), second (b.) and third (c.) MD replica.

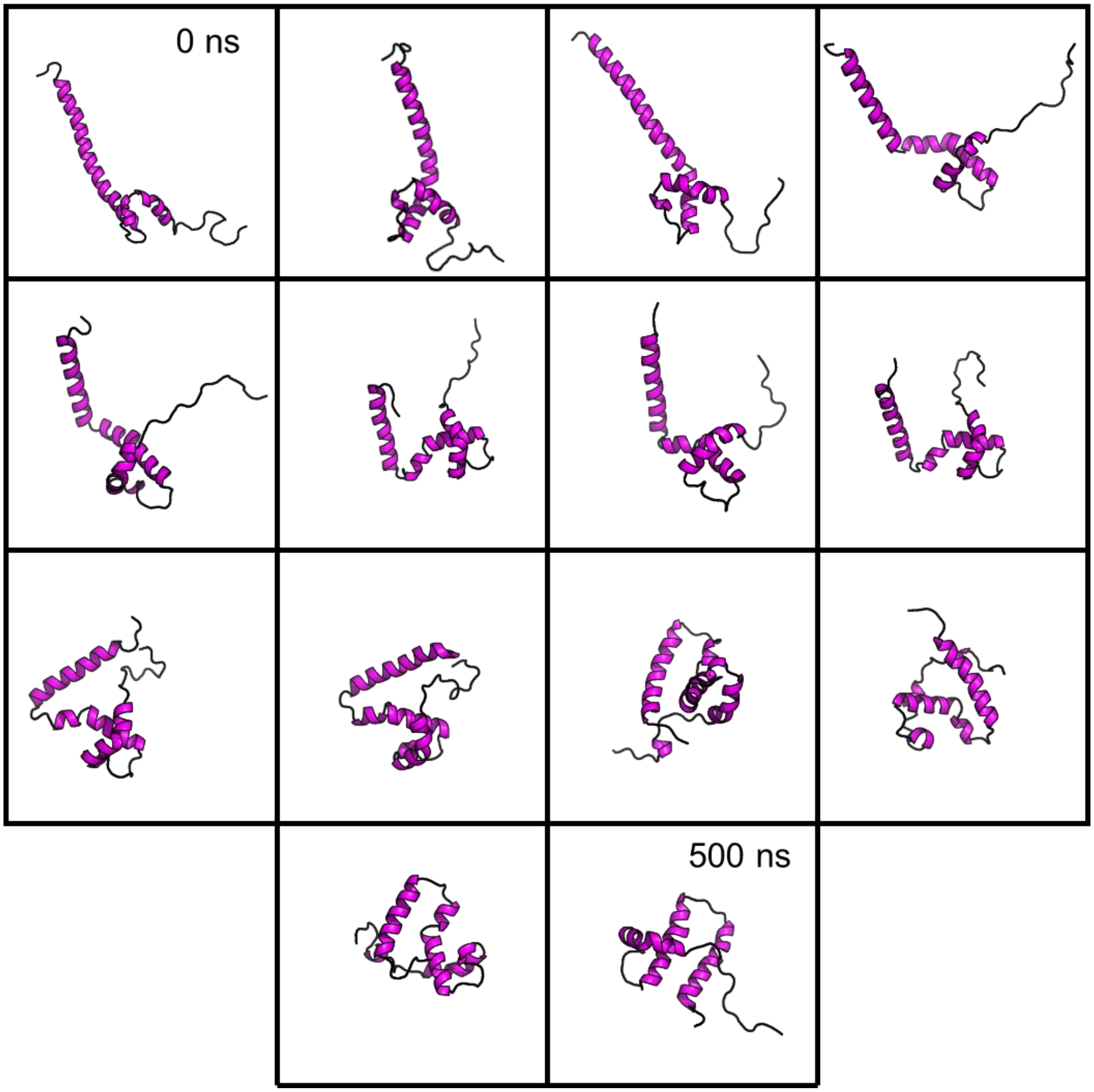

Figure S11. Conformations Sampled in MD run 1. Snapshots of the first MD simulation of MAX.

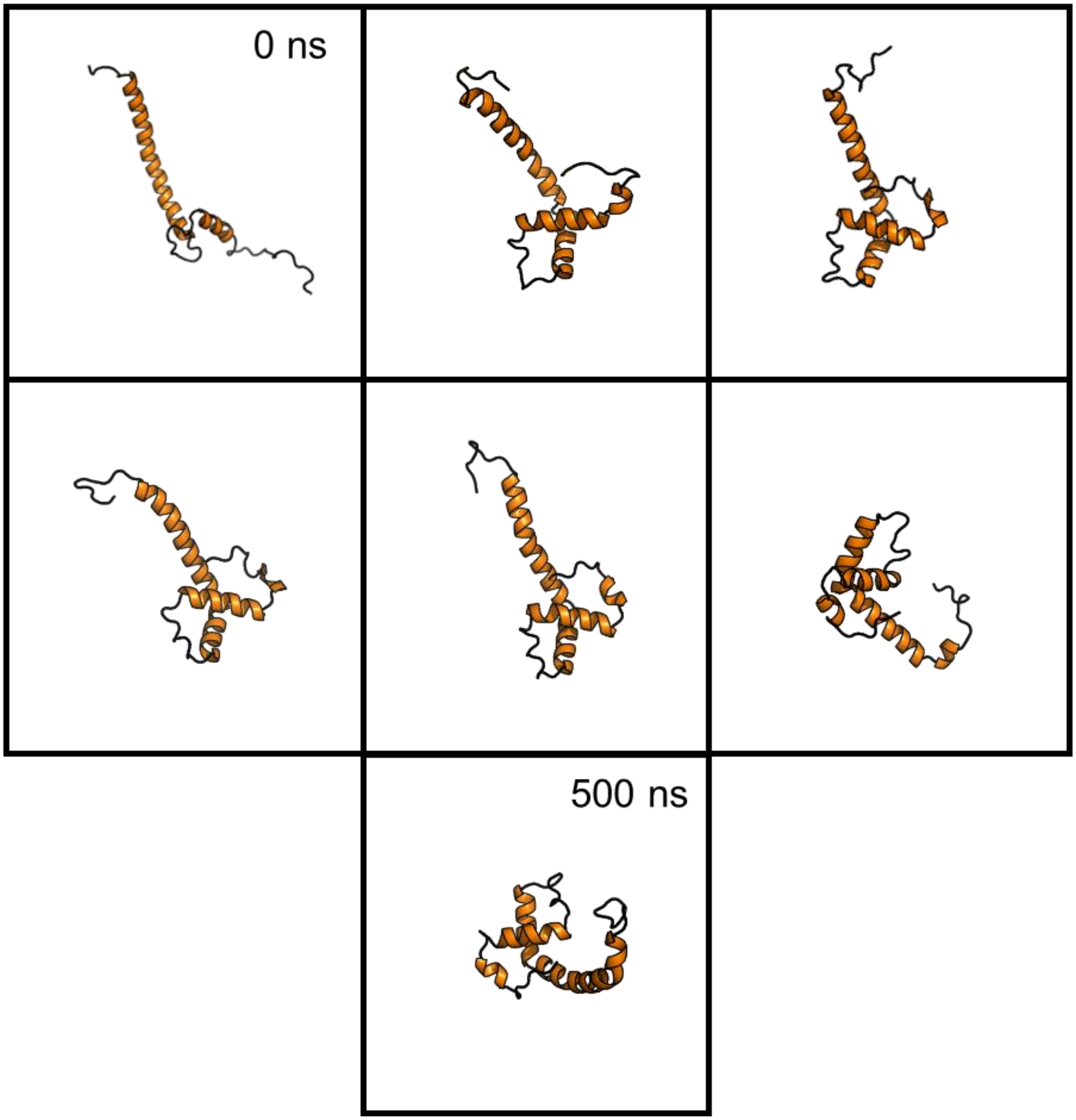

Figure S12. **Conformations Sampled in MD run 2.** Snapshots of the second MD simulation of MAX.

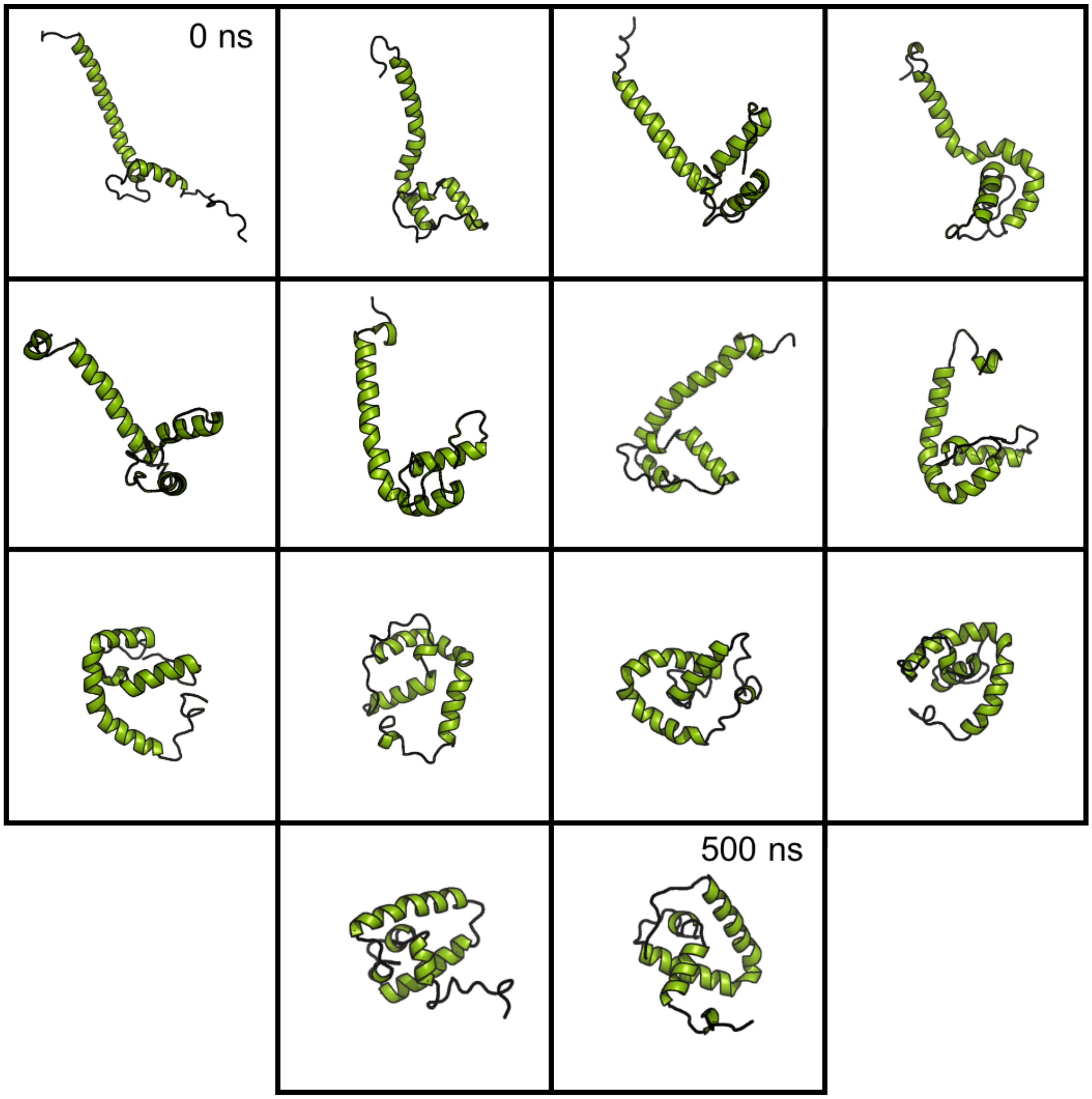

Figure S13. **Conformations Sampled in MD run 3.** Snapshots of the third MD simulation of MAX.

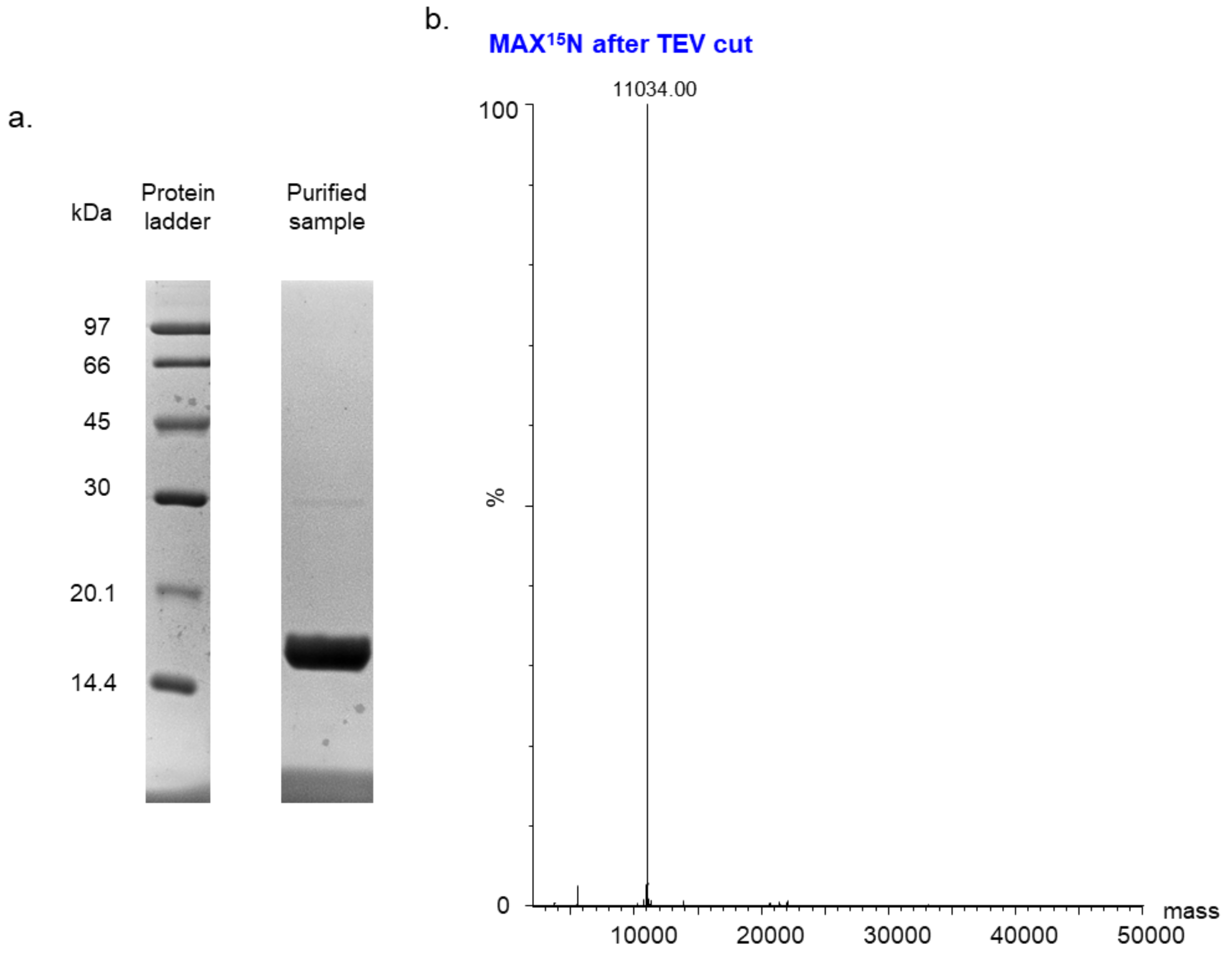

Figure S14. **Protein Purification.** Elution profile of MAX ^{15}N after affinity chromatography purification. a) SDS-PAGE of purified His-tagged MAX (^{15}N labeled). The protein mass should be approximately 15 kDa, while the expected uncut protein has a MW of ~13.99 kDa. The 1 kDa discrepancy is due to the high net charge of MAX even in the presence of SDS. b) Deconvolute mass spectrum of a purified MAX ^{15}N sample. The mainly detected mass (11.034 ± 0.05 kDa) is consistent with the expected mass of a homogeneously labeled MAX ^{15}N (11.085 kDa).

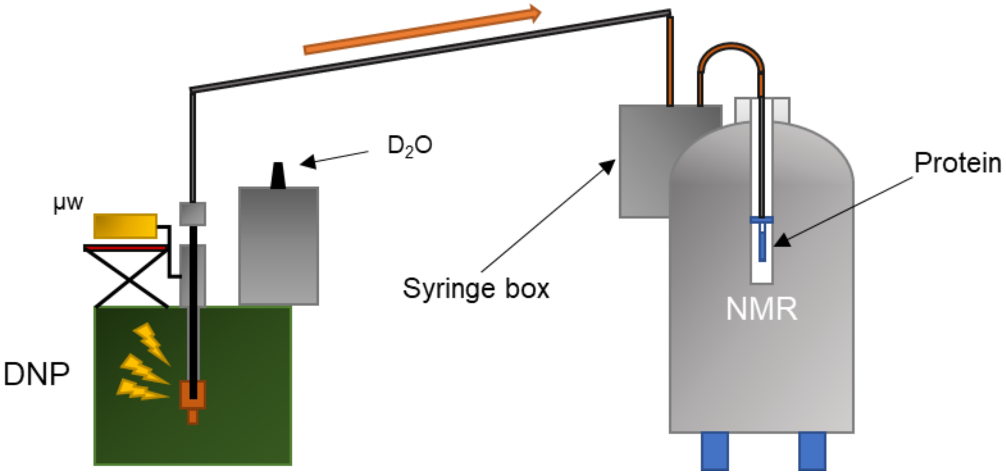

Figure S15. **DDNP Setup.** Schematic representation of the DDNP set up at the University of Vienna. The DNP sample containing TEMPOL is hyperpolarized in a cryostat by microwave irradiation. After polarization, hot D_2O at high pressure is injected into the polarizer, dissolving the sample that is consequently transferred through a magnetic tunnel into a "syringe box" injection system that controls the injected volume.

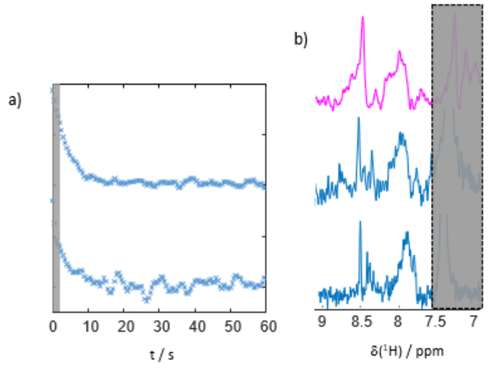

Figure S16. Reproducibility checks. Reproductions of the DDNP experiments. a) Signal intensity decay for two repetitions. b) Spectra obtained from the repetition experiments (blue) compared to the one in the main text (magenta). The grey boxes indicate regions excluded from the analysis as in the main text.

Table S1. Sparta+ results. Averaged signal intensity in dependence of the chemical shift from the SPARTA+ predictions for the first, second and third MD simulation run.

first simulation			second simulation			third simulation	
CS	***S / a.u.***		***CS***	***S / a.u.***		***CS***	***S / a.u.***
6.800	0.000		6.740	0.000		6.740	0.042
6.828	0.000		6.769	0.000		6.769	0.000
6.857	0.120		6.798	0.000		6.798	0.000
6.885	0.000		6.827	0.000		6.827	0.000
6.913	0.000		6.856	0.000		6.856	0.000
6.941	0.000		6.884	0.000		6.884	0.083
6.970	0.000		6.913	0.000		6.913	0.000
6.998	0.000		6.942	0.000		6.942	0.000
7.026	0.000		6.971	0.000		6.971	0.000
7.055	0.000		7.000	0.000		7.000	0.000
7.083	0.000		7.029	0.000		7.029	0.083
7.111	0.000		7.058	0.000		7.058	0.000
7.139	0.000		7.087	0.000		7.087	0.000
7.168	0.000		7.116	0.000		7.116	0.000
7.196	0.000		7.144	0.000		7.144	0.167
7.224	0.000		7.173	0.000		7.173	0.000
7.253	0.080		7.202	0.000		7.202	0.292
7.281	0.000		7.231	0.000		7.231	0.083
7.309	0.000		7.260	0.043		7.260	0.083
7.337	0.040		7.289	0.000		7.289	0.042
7.366	0.000		7.318	0.000		7.318	0.167
7.394	0.040		7.347	0.000		7.347	0.083
7.422	0.120		7.376	0.000		7.376	0.208
7.451	0.040		7.404	0.000		7.404	0.125
7.479	0.240		7.433	0.130		7.433	0.417
7.507	0.080		7.462	0.000		7.462	0.375
7.535	0.120		7.491	0.000		7.491	0.500
7.564	0.160		7.520	0.000		7.520	0.333
7.592	0.000		7.549	0.304		7.549	0.208
7.620	0.240		7.578	0.174		7.578	0.458
7.648	0.480		7.607	0.174		7.607	0.333
7.677	0.240		7.636	0.391		7.636	0.625
7.705	0.440		7.664	0.261		7.664	0.333
7.733	0.200		7.693	0.565		7.693	0.333
7.762	0.320		7.722	0.261		7.722	0.292
7.790	0.400		7.751	0.870		7.751	0.708

7.818	0.320		7.780	0.478		7.780	0.625
7.846	0.200		7.809	0.783		7.809	0.792
7.875	0.600		7.838	0.565		7.838	0.500
7.903	0.280		7.867	0.609		7.867	0.458
7.931	0.240		7.896	0.391		7.896	0.583
7.960	0.840		7.924	0.391		7.924	0.458
7.988	0.360		7.953	1.000		7.953	0.458
8.016	0.560		7.982	0.652		7.982	0.292
8.044	0.560		8.011	0.870		8.011	0.583
8.073	0.640		8.040	0.522		8.040	0.458
8.101	0.680		8.069	0.739		8.069	0.125
8.129	0.680		8.098	0.652		8.098	0.542
8.158	0.760		8.127	0.870		8.127	0.417
8.186	0.360		8.156	0.652		8.156	0.417
8.214	0.520		8.184	0.522		8.184	0.583
8.242	0.560		8.213	0.652		8.213	0.458
8.271	0.480		8.242	0.174		8.242	0.500
8.299	0.720		8.271	0.783		8.271	0.500
8.327	0.400		8.300	0.391		8.300	0.375
8.356	0.520		8.329	0.652		8.329	1.000
8.384	0.320		8.358	0.565		8.358	0.958
8.412	0.160		8.387	0.435		8.387	0.625
8.440	0.400		8.416	0.130		8.416	0.292
8.469	1.000		8.444	0.522		8.444	0.583
8.497	0.600		8.473	0.304		8.473	0.375
8.525	0.720		8.502	0.957		8.502	0.458
8.554	0.520		8.531	0.478		8.531	0.458
8.582	0.320		8.560	0.522		8.560	0.292
8.610	0.600		8.589	0.174		8.589	0.083
8.638	0.440		8.618	0.478		8.618	0.292
8.667	0.480		8.647	0.609		8.647	0.375
8.695	0.360		8.676	0.391		8.676	0.333
8.723	0.440		8.704	0.696		8.704	0.250
8.752	0.160		8.733	0.304		8.733	0.125
8.780	0.160		8.762	0.217		8.762	0.333
8.808	0.320		8.791	0.000		8.791	0.292
8.836	0.200		8.820	0.087		8.820	0.125
8.865	0.360		8.849	0.261		8.849	0.167
8.893	0.000		8.878	0.174		8.878	0.125
8.921	0.160		8.907	0.304		8.907	0.000
8.949	0.080		8.936	0.174		8.936	0.000

8.978	0.200		8.964	0.000		8.964	0.042
9.006	0.000		8.993	0.043		8.993	0.167
9.034	0.120		9.022	0.130		9.022	0.125
9.063	0.120		9.051	0.261		9.051	0.000
9.091	0.240		9.080	0.174		9.080	0.000
9.119	0.280		9.109	0.261		9.109	0.000
9.147	0.080		9.138	0.000		9.138	0.000
9.176	0.000		9.167	0.087		9.167	0.000
9.204	0.000		9.196	0.000		9.196	0.000
9.232	0.040		9.224	0.087		9.224	0.000
9.261	0.000		9.253	0.043		9.253	0.000
9.289	0.000		9.282	0.000		9.282	0.000
9.317	0.000		9.311	0.087		9.311	0.000
9.345	0.000		9.340	0.000		9.340	0.000
9.374	0.000		9.369	0.000		9.369	0.000
9.402	0.000		9.398	0.000		9.398	0.083
9.430	0.000		9.427	0.000		9.427	0.000
9.459	0.000		9.456	0.000		9.456	0.000
9.487	0.000		9.484	0.000		9.484	0.000
9.515	0.000		9.513	0.000		9.513	0.000
9.543	0.000		9.542	0.000		9.542	0.000
9.572	0.000		9.571	0.000		9.571	0.000
9.600	0.000		9.600	0.000		9.600	0.000
